# Stratum corneum hydration levels are negatively correlated with HbA1c levels in the elderly Chinese

**DOI:** 10.1111/1753-0407.70022

**Published:** 2024-10-27

**Authors:** Qingsong Lai, Xiaohua Wang, Zebin Lai, Yulin Lai, Li Ye, Sha Liu, Bin Yang, Mao‐Qiang Man

**Affiliations:** ^1^ Department of Dermatology Medical Center for Public Health of Puning Puning City China; ^2^ Dermatology Hospital Southern Medical University Guangzhou China; ^3^ Department of Dermatology Maternal and Child Health Care Hospital of Punning Puning City China

**Keywords:** diabetes, HbA1c, skin surface pH, stratum corneum hydration, transepidermal water loss

## Abstract

**Highlights**

- Stratum corneum hydration levels are negatively correlated with HbA1c levels and positively correlated with skin surface pH.
- Individuals with type 2 diabetes display lower levels of stratum corneum hydration.
- Because low stratum corneum hydration levels can increase circulating levels of proinflammatory cytokines, which are linked to the pathogenesis of type 2 diabetes, improvement in stratum corneum hydration can be an alternative approach in the management of type 2 diabetes.

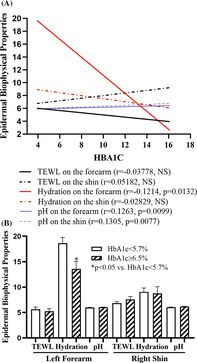

To the Editor

Alterations in systemic conditions can reflect the changes in epidermal biophysical properties. Type 2 diabetes is the condition that body does not respond to insulin normally, resulting in an elevation in blood glucose levels. High glucose levels inhibit keratinocyte proliferation, while stimulating differentiation in high calcium‐treated keratinoctyes.^1^, ^2^ Moreover, high glucose increases cytokine secretion and oxidative stress in keratinocytes.^3^ Because both keratinocyte differentiation and cytokines can affect epidermal function,^4^, ^5^ individuals with diabetes can display altered epidermal functions, manifested by the changes in epidermal biophysical properties. Indeed, several studies demonstrate the changes in epidermal functions in subjects with either type 1 or type 2 diabetes.^6^ However, the results are inconsistent, in part due to the small sample size. Here we compared the epidermal biophysical properties between subjects with versus without type 2 diabetes (HbA1c ≥6.5% vs. HbA1c <5.7%) in a larger cohort of elderly Chinese.

This study was carried out between October 2023 and March 2024. All participants were enrolled from the outpatient clinic of the Medical Center for Public Health of Puning, Guangdong, China. Because the prevalence of type 2 diabetes is higher in people aged 65 years and older,^7^ only individuals aged ≥65 years were enrolled in this study. All participants were without current or a history of atopic dermatitis or psoriasis or diseases with abnormal keratinization. Except for normal bathing or showering, no topical products were applied to the forearm and the shin within 24 h prior to the measurements of epidermal biophysical properties. Epidermal biophysical properties, including TEWL and stratum corneum hydration, were measured with GPskin Barrier® (GPower Inc., Seoul, South Korea), while skin surface pH was measured with a portable skin pH meter (Nate Instrument, Suzhou, Jiangsu, China) on the flexor of the left forearm and the right shin. The measurement sites were 15 cm above the wrist and 10 cm below the knee. All measurements were performed in a designated room at a temperature of 18–22°C and relative humidity of 55%–65%. This study was approved by the institutional review board of the Dermatology Hospital of Southern Medical University (GDDHLS2021025). Written informed consent was obtained from the participants prior to the study. The Mann–Whitney test was used to determine the significance between individuals with hemoglobin A1c (HbA1c) <5.7% and ≥6.5%, while pearson correlation analysis was used to determine significances of the association between HbA1c and epidermal biophysical properties. Data are expressed as mean ± SEM. *p* values are indicated in the tables, figures or figure legends.

A total of 416 subjects, including 201 males and 215 females, aged 65 to 98 years, were enrolled in this study (Table 1). There were a total of 92 individuals with HbA1c ≥ 6.5% and 202 subjects with HbA1c < 5.7%. The age of subjects with HbA1c ≥ 6.5% was comparable to that with HbA1c < 5.7% (71.96 ± 0.50 vs. 73.76 ± 0.47). Although no significant correlation between age and epidermal biophysical properties was observed in this cohort, stratum corneum hydration levels on the forearm were negatively correlated with HbA1c levels (Figure 1A; *p* < 0.05). In contrast, skin surface pH was positively correlated with HbA1c levels (Figure 1A; *p* < 0.01). But blood fast glucose levels were not correlated significantly with any of epidermal biophysical properties (*p* > 0.05 for all). As shown in Figure 1B, subjects with HbA1c levels of ≥6.5% exhibited significantly lower stratum corneum hydration levels on the forearm (13.59 ± 1.49 vs. 18.63 ± 1.14, *p* = 0.0215). Although skin surface pH was positively correlated with HbA1c, the pH level did not differ significantly between individuals with HbA1c ≥ 6.5% versus <5.7% (6.04 ± 0.05 vs. 5.64 ± 0.44 on the forearm; 6.17 ± 0.09 vs. 6.04 ± 0.04 on the shin) (Figure 1B). Collectively, these results demonstrate a decreased stratum corneum hydration levels in individuals with type 2 diabetes compared with those without type 2 diabetes.

**TABLE 1:** Demographic characteristics of participants.

Gender	*N*	Age (year)	Fasting blood glucose (mmol/L)	HbA1c (%)	BMI (kg/m^2^)
Males	201	73.77 ± 0.46	7.84 ± 0.21**	5.90 ± 0.08*	22.87 ± 0.26**
Females	215	72.82 ± 0.43	8.94 ± 0.25	6.18 ± 0.10	24.89 ± 0.26
Total	416	73.28 ± 0.31	8.41 ± 0.17	6.04 ± 0.07	23.92 ± 0.19

*Note*: Data are expressed as mean ± standard error of mean.

* *p* = 0.0077;

** *p* <0.0001 versus females.

**FIGURE 1:**
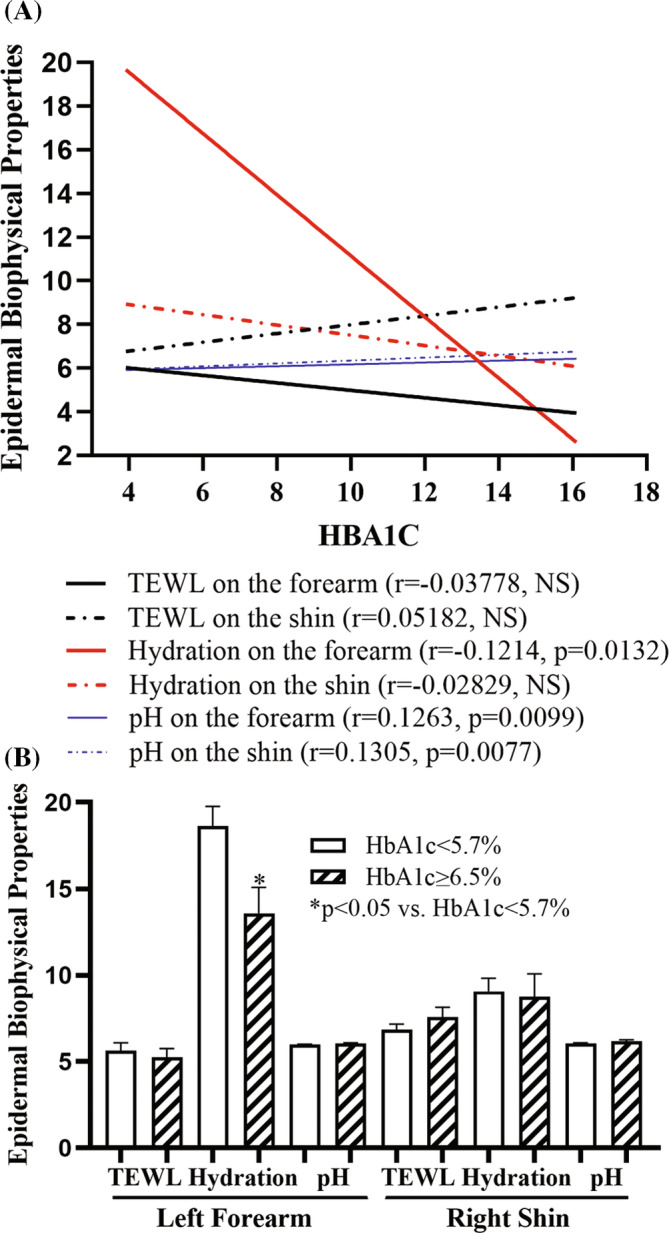
Correlation of HbA1c with epidermal biophysical properties. (A) Correlation of HbA1c with epidermal biophysical properties (*N* = 416). (B) Comparison of epidermal biophysical properties in individuals with HbA1c levels < 5.7% versus ≥6.5%. *N* = 92 for individuals with HbA1c ≥ 6.5%; *N* = 202 for individuals with HbA1c < 5.7%. Significances are indicated in the figures.

The present study showed that HbA1c levels are negatively correlated with stratum corneum hydration, while positively correlating with skin surface pH in the elderly. Moreover, individuals with higher HbA1c levels (≥6.5%) exhibited significantly lower stratum corneum hydration levels than those with normal HbA1c levels (<5.7%), which is consistent with previous finding in subjects with type 2 diabetes.^8^ It is worth noting that the stratum corneum hydration levels were lower in the present study than that previously reported.^9^ It is no surprise that the subjects in the previous studies were much younger than that in the present study (33.91 ± 7.17 [SD] vs. 73.28 ± 6.39 [SD]). It is well‐known that stratum corneum hydration levels are lower in aged versus young humans.^10^ Moreover, individuals with diabetes can display a lower level of stratum corneum hydration.^10^ The average HbA1c and fasting blood glucose levels in participants of the present study were far above normal range. Hence, both the subjects' age and higher blood glucose can account for discrepancy in the stratum corneum hydration levels between the present and the previous studies. The reduced stratum corneum hydration levels in individuals with type 2 diabetes are possibly attributable to the decreased stratum corneum lipid contents,^8^ key determinator for stratum corneum hydration.^11^, ^12^ Although it is not clear whether low stratum corneum hydration increases HbA1c or vice versa, low stratum corneum hydration can negatively impact type 2 diabetes. Previous study showed that stratum corneum hydration levels are inversely correlated with serum levels of pro‐inflammatory cytokines.^13^ Accordingly, improvement in stratum corneum hydration lowers proinflammatory cytokine levels in both the skin and circulation in humans and mice.^14^, ^15^, ^16^ Individuals with type 2 diabetes, manifested by elevated blood HbA1c and glucose levels, display high circulating levels of proinflammatory cytokines,^17^ which play a crucial role in the pathogenesis of type 2 diabetes.^18^ Thus, reduction in the stratum corneum hydration levels can induce and exacerbate type 2 diabetes in the elderly. However. Further studies are needed to elucidate the interaction between stratum corneum hydration levels and type 2 diabetes.

## CONCLUSIONS

Stratum corneum hydration levels are negatively correlated with serum HbA1c levels in the elderly. Because low stratum corneum hydration levels can increase circulating levels of proinflammatory cytokines, which are linked to the pathogenesis of type 2 diabetes, improvement in stratum corneum hydration can be an alternative approach in the management of type 2 diabetes.

## AUTHOR CONTRIBUTIONS

Qingsong Lai, Xiaohua Wang, Zebin Lai, Yulin Lai, Li Ye and Sha Liu performed the experiments, collected the data, and critically reviewed the manuscript. Bin Yang coordinated and supervised the study and critically reviewed the manuscript for important intellectual content. Mao‐Qiang Man conceptualized and designed the study, drafted the initial manuscript, and revised the manuscript. All authors approved the final manuscript as submitted and agree to be accountable for all aspects of this work.

## CONFLICT OF INTEREST STATEMENT

The authors declare no conflicts of interest.

## INFORMED CONSENT

Written informed consent was obtained from the participants prior to the study.

## Data Availability

Data supporting this report are available from QL and MQM.
